# Knocking down the obstacles to functional genomics data sharing

**DOI:** 10.1038/sdata.2017.19

**Published:** 2017-03-01

**Authors:** Kaylene J. Simpson, Jennifer A. Smith

**Affiliations:** 1 Victorian Centre for Functional Genomics, Peter MacCallum Cancer Centre, Melbourne 3000, Australia; 2 The Sir Peter MacCallum Department of Oncology, University of Melbourne, Parkville 3010, Australia; 3 ICCB-Longwood Screening Facility, Harvard Medical School, Boston, Massachusetts 02115, USA

**Keywords:** High-throughput screening, Inhibitory RNA techniques

## Abstract

This week, *Scientific Data* published a collection of eight papers that describe datasets from high-throughput functional genomics screens, primarily utilizing RNA interference (RNAi). The publications explore host-pathogen dependencies, innate immune response, disease pathways, and cell morphology and motility at the genome-level. All data, including raw images from the high content screens, are publically available in PubChem BioAssay, figshare, Harvard Dataverse or the Image Data Resource (IDR). Detailed data descriptors enable use of these data for analysis algorithm design, machine learning, data comparisons, as well as generating new scientific hypotheses.

## Comment

Over the past decade, the term ‘functional genomics’ has become synonymous with large, often genome-scale, interrogation of gene function in a highly systematic and unbiased manner, principally relying on laboratory automation and often coupled with quantitative high-content phenotypic imaging. Cell-based experiments are miniaturized to microplate format (96-, 384- or 1536-well) and optimized, when possible, using well-defined positive and negative controls. The goal is to develop biologically relevant, robust assays. These efforts are intensive in terms of the cost and time investment required to develop, optimize and conduct them, as well as to perform subsequent data analysis and validation experiments. This, however, has not diminished the interest in, and value of, RNA interference (RNAi) as a discovery tool to explore a broad range of biological questions.

Arrayed RNAi screens generate a substantial amount of data. In its simplest form, a genome-scale viability screen performed in duplicate that relies on a plate reader for assay readout generates more than 40,000 data points. At the other end of the spectrum, a full-genome high-content imaging screen utilizing multiple fluorescent channels and capturing a large number of cellular features will produce millions of data points. Extensive statistical analysis is required to interpret such datasets and, historically, the ‘screen’ portion of a scientific publication is relegated to a supplemental figure, with both the data and methodology behind it inadequately described, thus preventing data reuse. It is vital that these screen data and their accompanying methodologies be made available to the scientific community in a usable format. Thus far, several obstacles have hindered this. While genome-scale arrayed RNAi screens have been performed and published for over a decade, there have been relatively few public data repositories. GenomeRNAi, initially described in 2007 by Michael Boutros and colleagues at DKFZ, was a pioneering database in this field^1^. Yet, the majority of datasets have been acquired from publications and thus suffered from a lack of adequate author-provided descriptions and, at least for mammalian cell-based screens, frequently contained only a subset of the data. Several years ago the NCBI’s PubChem BioAssay^2^, an established repository for compound screening data, expanded to host RNAi screening data. Commercial vendors released their siRNA duplex sequence information, specified as substance identifiers, thus enabling scientists to deposit both raw and analyzed numerical data into the BioAssay standardized format portal. Even with this advance, however, reuse was hindered by the lack of a detailed description of how data were generated and analyzed. Data usability was also impeded by the lack of access to the corresponding raw images, thus preventing re-analysis and extraction of additional parameters.

This week, *Scientific Data* launched a collection of eight papers describing high-throughput functional genomics screens, exploring diverse biological processes at the genome-level, from host-pathogen dependencies to cell morphology and motility (http://www.nature.com/sdata/collections/funcgenom). Each contribution represents a concrete example of progress in overcoming the above-mentioned obstacles. Screen data are openly available via public data repositories, and the associated data records are clearly described in each publication. Notably, three of the screens relied on high-content imaging^3–5^ with all raw images made available in either figshare, Harvard Dataverse or the Image Data Resource (IDR; based on the Open Microscopy Environment^6^), demonstrating the feasibility of effectively describing and sharing data even in these challenging cases.

The publications within this collection represent recent functional genomics screening efforts and, for many, this is the first time they have been described. Four of the contributions describe siRNA^7–9^ or miRNA mimic and inhibitor^10^ screens with relatively straightforward plate reader-based outputs. The data are provided through PubChem BioAssay^2^. Even with one or two data points per well, the complexity of the screen designs and data analysis creates substantial variation that requires in depth description. The primary screening protocols, raw and analyzed data, as well as data analysis methodologies and hit prioritization are well described. These publications delineate secondary and tertiary screens that were conducted to confirm positives, eliminate off-target effects and determine specificity. The use of multiple siRNA reagents in the primary and secondary screens conducted in Iain Fraser’s laboratory^7,8^ are informative, as scientists now have a variety of strategies to address off-target effects in RNAi screens, and these large-scale siRNA duplex comparisons are essential in designing and testing analysis algorithms to predict off-target effects^11–13^. Validation also extended to additional, complementary genomics approaches. For example, Wu *et al.* ^9^ present a valuable comparison of results from RNAi-mediated knockdown and CRISPR/Cas9-mediated knockout, and Sun *et al.* ^8^ include a comparison to transcriptomics data.

Arrayed, high-throughput screening is particularly amenable to quantitative high-content imaging, but, as described above, data-sharing is more challenging as a consequence of the vast amount of data, size of raw images, variables in image acquisition and analysis, as well as the multiparametric nature of the data. This collection includes three examples of such screens, each focusing on distinct cellular phenotypes. The authors have addressed what has historically been lacking with publications of these kinds of screens—inclusion of all raw image files and a clear explanation of the entire screening protocol. Vargas and colleagues knocked down the kinome in triple negative breast cancer cell lines, then deep-phenotyped the siRNA-transfected cells, quantifying 127 different features as well as YAP/TAZ localization^4^. A binning strategy was then used to sub-classify the features into 5 distinct shapes. All images are available via IDR. Vascular dynamics was the focus of the genome-wide siRNA screen performed by Williams *et al.* ^3^. They utilized a scratch-wound healing assay to quantify cell motility in lymphatic vascular endothelial cells, with secondary screens expanding to blood endothelial cells; this high content screen enabled determination of core, conserved migration genes and cell line dependent targets. All images are available in figshare and the analyzed data are available in PubChem BioAssay. In contrast to the above two transfection-based screens, Ketteler and team relied on electroporation to deliver siRNAs targeting the human kinome into umbilical vein endothelial cells^5^. Image analysis quantified Weibel-Palade Body size, nuclei, the trans-Golgi network and plasma membrane staining. They developed a detailed analytical pipeline to integrate all cellular phenotypes. The raw images and data records are publically available at the Harvard Dataverse repository.

In contrast to the RNAi screens described above, Pettitt and colleagues conducted a forward-genetic screen^14^. They performed a genome-wide barcoded transposon screen to determine what mutations caused sensitivity to common cancer drugs in haploid murine embryonic stem cells. All scripts have been shared in GitHub and all FASTQ files are deposited in figshare, enabling re-use for a variety of applications.

Functional genomics datasets are a valuable resource to optimize new genomics tools, refine informatics approaches and improve machine learning. Re-analysis of screen data and raw images is an effective approach for hypothesis generation and identification of novel targets. While publications will no doubt continue to focus on a pathway or several targets identified in a large-scale screen, this requires extensive secondary and tertiary analysis, generally with different cell lines, biological assays, and validation with complementary approaches. The time investment is enormous. To share this abundance of data prior to or in conjunction with a more detailed publication focused on a specific aspect of the screen provides the research community with an opportunity to advance our knowledge, decrease duplicated efforts and conserve basic science resources.

## Additional Information

**How to cite this article**: Simpson, K. J. & Smith, J. A. Knocking down the obstacles to functional genomics data sharing. *Sci. Data* 4:170019 doi: 10.1038/sdata.2017.19 (2017).

**Publisher’s note**: Springer Nature remains neutral with regard to jurisdictional claims in published maps and institutional affiliations.
